# Evidence of cell cycle re-entry in post-mitotic, terminally differentiated feline neurons

**DOI:** 10.1007/s00418-022-02112-1

**Published:** 2022-05-12

**Authors:** Karolina Wisnet, Christoph H. F. Payer, Barbara Bauder, Angelika Url

**Affiliations:** Institute of Pathology, Department of Pathobiology, Vetmeduni Vienna, Veterinärplatz 1, 1210 Vienna, Austria

**Keywords:** Cat, Neuronal cell cycle, Parvovirus, pRb, Sox2

## Abstract

Parvovirus infections in dogs and cats are restricted to highly mitotically active tissues, predominantly to the epithelium of the gastrointestinal tract and, in cases of prenatal infections in cats, also to Purkinje cell neuroblasts. The evidence of parvovirus-infected mature feline neurons gave rise to reconsider the dogma of post-mitotically fixed and terminally differentiated neurons in the adult central nervous system. To elucidate the postulated capability of certain terminally differentiated feline neurons to re-enter the cell cycle, immunohistochemical double labeling using the transcription factor Sox2 and the tumor suppressor and cell cycle regulator retinoblastoma protein in its phosphorylated state (pRb) was performed. Formalin-fixed and paraffin-embedded brain tissue negative for parvovirus-antigen from 14 cats was compared to brain tissue from 13 cats with immunohistochemically confirmed cerebral parvovirus infection; the 27 cats were aged between 50 days of gestation (E50) and 5 years. Both groups revealed nuclear Sox2 and pRb immunosignals in numerous neurons, suggesting a more active state than mature neurons should have. Accordingly, parvovirus is not exclusively involved in the reactivation of the cell cycle machinery in those post-mitotic, terminally differentiated feline neurons.

## Introduction

Based on the single-stranded DNA genome of parvoviruses, parvovirus replication depends on cellular DNA replication mechanisms and mitotically active host cells; for initiation of DNA replication, the host cell must go through the S-phase (Berns 1990). Mature neurons in turn, although nowadays believed to be capable of re-entering the cell cycle, seem not to overcome G1/S transition (Nagy 2000; Zhang et al. 2020). Thus, S-phase-related DNA polymerase, which is required for the synthesis of essential double-stranded intermediates prior to parvovirus transcription and translation (Berns 1990), should not be active in mature neurons. However, contrary to the hypothesis of permanent G0-restriction of mature neurons (Nagy 2000; Zhang et al. 2020), there is evidence of parvovirus-antigen and viral mRNA translation and replication in mature neurons of cats (Garigliany et al. 2016; Url et al. 2003), as well as the expression of S-phase proteins in neurons of cats with naturally occurring panleukopenia (Poncelet et al. 2016).

In Alzheimer’s and Parkinson’s disease or stroke, there is accumulating evidence of re-expression and activation of cell cycle proteins in dying neurons and of interactions between cell cycle machinery in post-mitotic neurons and both apoptosis and DNA repair (Kruman 2004; Zhang et al. 2020). Hence, we intended to test the hypothesis of whether parvovirus-infected (dying) feline neurons are forced to re-enter the cell cycle.

To immunohistochemically clarify the cell cycle activity of terminally differentiated feline neurons in conjunction with neuronal parvovirus infections retinoblastoma protein (pRb) and Sox2 seemed promising; Sox2, as it shows next to its classical role in developmental processes and reprogramming capacities (Julian et al. 2017; Karow et al. 2018) interactions with the cell cycle regulator cyclin D (Han et al. 2014; Swistowska et al. 2019), and pRb as it plays a key role in cell cycle progression (Giacinti and Giordano 2006; Mittnacht 1998; Weinberg 1995) and neuronal survival (Andrusiak et al. 2012).

In detail, the phosphorylation sites S608 and S795 used in this study are known to interact with E2F (Rubin 2013), another crucial player within the cell cycle machinery concerning checkpoint G1/S (Giacinti and Giordano 2006; Mittnacht 1998; Zhang et al. 2020). Furthermore, S795 is presumably phosphorylated by neurotoxicity-mechanisms in response to angiotensin II (MAP kinase pathway), e.g., in HIV infections (Rubin 2013), and therefore possibly also in parvovirus infected cats.

## Materials and methods

### Specimens

We investigated 27 brains of euthanized cats or cats that died from severe illnesses and were submitted for necropsy (Institute of Pathology, Vetmeduni Vienna). Group A consisted of 14 immunohistochemically parvovirus-antigen negative brains from cats aged from E50 to 12 months, whereas group B included 13 parvovirus-antigen positive brains from cats between 6 weeks and 5 years (Table 1). None of the 27 brains showed neuropathological alterations, except pyogranulomatous leptomeningoencephalitis due to feline infectious peritonitis (FIP) in two cats of group A; besides two brains (one of each group) proven immunohistochemically positive for feline leukemia virus (FeLV), all other brains were negative for feline herpesvirus type 1 and FeLV. In a previous study, all brains of group A revealed Sox2 immunosignals (Sox2^+^) in mature neurons (Payer 2018).

**Table 1:** Details of the 27 cats included in this study

Group A^a^			Group B^b^		
Age	Sex	Diagnosis	Age	Sex	Diagnosis
E50	M	Abortion	6 weeks	F	Panleukopenia
2 days	M	Septicemia	6 weeks	M	Panleukopenia
2 weeks	F	Septicemia	2 months	NK	Panleukopenia
1 month	NK	NK	2.5 months	M	Panleukopenia
3 months	F	FIP	3 months	F	Renal dysplasia, panleukopenia
4 months	F	Cerebral FIP	3 months	F	Panleukopenia
5 months	M	FIP	3.5 months	F	Demyelinating encephalopathy
6 months	M	Panleukopenia	4 months	F	Panleukopenia
7 months	M	Trauma	4.5 months	M	Hemorrhagic enteritis
8 months	M	Cerebral FIP	5 months	M	Anemia, cerebellar hypoplasia
9 months	NF	FIP	8 months	NM	Panleukopenia
10 months	F	FIP	3 years	M	Panleukopenia
11 months	M	Intoxication, trauma	5 years	NM	Panleukopenia
1 year	NM	Plant intoxication			

*E50* 50th embryonic day (50 days of gestation), *M* male, *F* female, *NM* neutered male, *NF* neutered female, *NK* not known, *FIP* feline infectious peritonitis

^a^Brain/neurons parvovirus-antigen negative

^b^Brain/neurons parvovirus-antigen positive

### Ethical approval

As the samples were collected for routine diagnostic purposes, no ethics committee approval was necessary. Compliance with federal law and good scientific practice is ensured.

### Immunohistochemistry

**Fig. 1:**
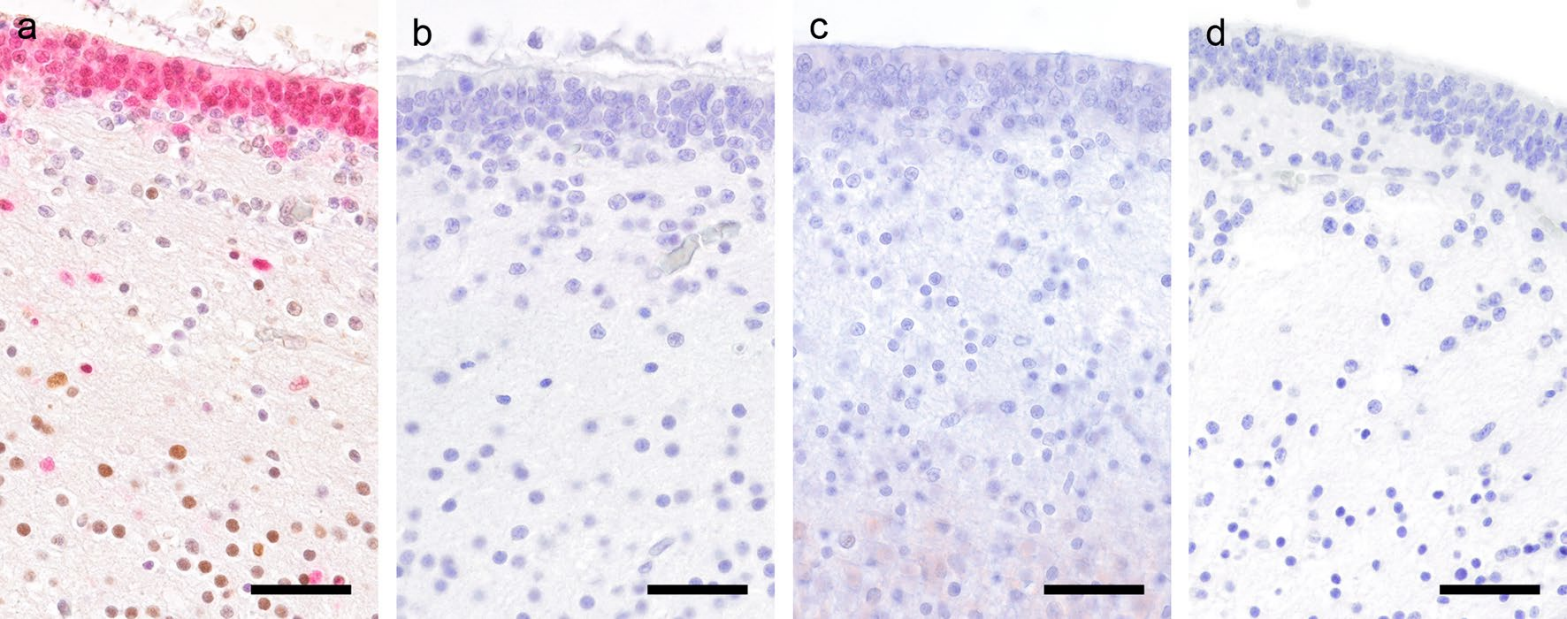
Positive and negative controls for immunohistochemistry. **a** Positive control for Sox2 (*red nuclei*) and pRb (*brown nuclei*) used in every double labeling run; note cytoplasmic reddish Sox2 signals within the germinal layer. **b** Lack of immunosignal within the isotype-control for Sox2 using IgG mouse as primary antibody. **c** Slight cytoplasmic immunosignal in the deeper layer within the isotype-control for pRb using IgG rabbit. Note the clear lack of nuclear signals. **d** Complete negative staining of the entire brain tissue by omitting the primary antibody. All panels: diencephalic region adjacent to lateral ventricles of cat E50,* bar* = 40 µm

With special attention to the lateral geniculate body, one of the main parvovirus replication sites within the feline brain (Url et al. 2003), 2-µm sections of formalin-fixed and paraffin-embedded tissue blocks taken at the level of the metathalamus, were deparaffinized, rehydrated, and heated to 97 °C for 20 min in citrate buffer (pH 6) for antigen retrieval. Briefly, double staining was performed on an Autostainer® (Lab Vision™ AS 360; Thermo Fisher Scientific, Waltham, MA, USA) as follows: Unspecific background staining due to endogenous peroxidase activity was blocked with UltraVision Hydrogen Peroxide Block (Thermo Fisher Scientific; incubation for 5 min), while background staining due to unspecific antibody binding was reduced by application of UltraVision Protein Block (Thermo Fisher Scientific; incubation for 10 min). Initially, the sections were incubated with the first primary antibodies (pRb^S608^ monoclonal rabbit, 1:200, ab172975, abcam®, Cambridge, UK or pRb^S795^ polyclonal rabbit, 1:200, ab47474, abcam®) for 30 min and were subsequently localized by an HRP-labeled polymer conjugated secondary antibody (BrightVision Poly-HRP-anti-rabbit IgG; ImmunoLogic, Duiven, Netherlands; incubation for 30 min). The polymer complex was visualized with diaminobenzidine (DAB Quanto Substrate System, Thermo Fisher Scientific; incubation for 5 min). Afterwards, those sections were incubated with the second primary antibody Sox2 (monoclonal mouse, 1:100, sc-365823, Santa Cruz Biotechnology, Dallas, TX, USA) for 30 min and conjugated with the appropriate detection system (BrightVision Poly-AP-anti-mouse IgG; ImmunoLogic; incubation for 30 min) before the polymer complex was visualized with ImmPACT Vector Red (Vector Laboratories, Burlingame, CA, USA; incubation for 20 min). After each step the slides were alternately washed with Tris-buffered saline with Tween™ (TBS; Thermo Fisher Scientific) and Aqua Dest. Subsequently, all sections were counterstained with hematoxylin, dehydrated and mounted (Neo-Mount®; Merck KGaA, Darmstadt, Germany). Additionally to the manufacturer’s proven specificity of the antibodies, we proved signal specificity on feline tissues by using proper negative and positive controls during the establishing process and in every immunohistochemical run, respectively (Fig. 1). Determination of non-specific bindings was proven by omitting the primary antibody, and by using isotype-specific immunoglobulins (IgG rabbit for pRb; IgG mouse for Sox2). Furthermore, as we applied Sox2 to mature neurons, we submitted the appropriate sequence (Sox2 (human) mapping to *3q26.33*, epitope mapping between amino acids 170–201; Sox-2 Datasheet, Santa Cruz Biotechnology, Dallas, TX, USA) to Basic Local Alignment Search Tool (BLAST); thus we could exclude unintentional cross-reactivity with feline neuron specific genes and with diverse Sox subclass factors, known to be involved in cell-fate processes of mature neurons, i.e., Sox4, Sox11, Sox14, and Sox21 (Julian et al. 2017). In order to establish pRb for feline tissue, human adenocarcinoma followed by feline gut, mammary gland carcinoma, and brain tissue were tested. As feline brain from cat E50 already served as a positive control for the routinely used Sox2 antibody, it subsequently was included in every double labeling run as positive control for Sox2 and pRb, respectively (Fig. 1a).

### Data analysis

Evaluation of immunosignals was performed using an Olympus BX51 microscope (Olympus Corporation, Tokyo, Japan) equipped with 60x/0.90 UPlan FL N dry and 100x/1.35 oil iris UPlan Apo objectives. For image acquisition, an Olympus UC90 CCD color camera (9.1 Mpx) with the corresponding software (Olympus cellSense 2.3 Life Science Imaging Software) was used. Pictures were brightness- and contrast-adjusted using Photoshop CS 12.1.

## Results and discussion

**Fig. 2:**
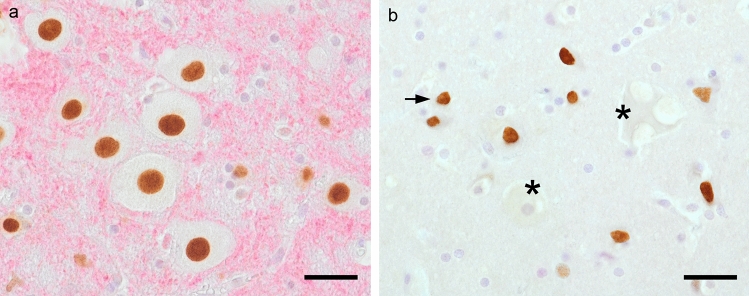
Immunohistochemistry with the monoclonal pRb^S608^ antibody. **a** Neurons of the lateral geniculate body of the 9-months-old group A cat showing specific brown nuclear signals in morphologically intact neurons. **b** Brownish immunosignals in shrinking (*arrow*) neurons of the 3-years-old group B cat, proven positive for neuronal parvovirus-antigen; note fading (*asterisks*) neurons. The* bar* in both panels = 30 µm

**Fig. 3:**
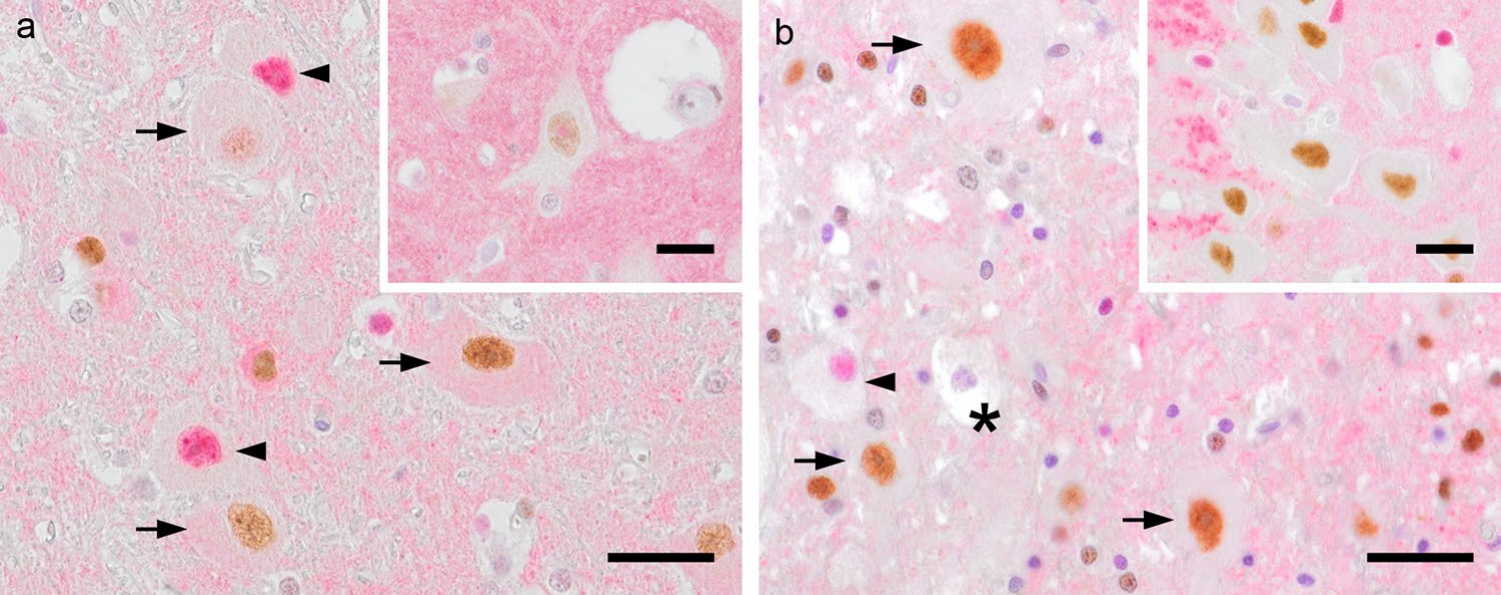
Immunohistochemistry using polyclonal pRb^S795^ and Sox2 antibodies. **a** Numerous mature neurons of the lateral geniculate body revealing pRb ^S795^ (*arrows*) and Sox2 (*arrowheads*) immunostaining in the 7-months-old cat of group A.* Inset*: A pRb^S795^-Sox2 double-stained cortical neuron of the 11-months-old group A cat; note brownish pRb^S795^ nucleus with a pinkish Sox2^+^ nucleolus. **b** Brown pRb^S795^ (*arrows*) and pink Sox2 (*arrowhead*) nuclei in the 5-months-old cat of group B, and an only slight Sox2^+^ nucleus within a fading neuron (*asterisk*).* Inset*: Strong pRb^S795^ signal in nuclei of numerous hippocampus neurons in the 5-years-old group B cat. The* bar* in both panels = 30 µm,* bar* in both insets = 20 µm

To determine whether parvovirus reactivates the cell cycle in feline terminally differentiated neurons, we applied immunohistochemistry to 27 cat brains with different neuronal parvovirus-antigen states using pRb^S608^, pRb^S795^, and Sox2 antibodies. All antibodies used gave rise to specific nuclear immunosignals within neurons of the lateral geniculate body (Figs. 2, 3) and adjacent gray matter structures (i.e., thalamic nuclei, hippocampus, cortex; Fig. 3a, b insets) in all cats.

### Immunosignals using pRb and Sox2 markers

Though all cats of both groups yielded positive immunosignals in numerous nuclei of morphologically mature neurons, the 2-weeks-old cat of group A revealed only light pRb^S608^ signals and was negative for pRb^S795^. In general, pRb^S608^ signals were much more intense than signals obtained with pRb^S795^. As the phosphorylation sites of the pRb markers used in the present study are known to directly interact and release E2F in late G1 (Giacinti and Giordano 2006; Mittnacht 1998; Rubin 2013; Zhang et al. 2020), the yielded immunosignals using those markers point onto activated mature neurons.

Further evidence of cell cycle activation in terminally differentiated feline neurons is proven, as Sox2, primarily expressed in neuronal progenitors (Encinas et al. 2006), was detected concomitantly with pRb in those neurons. Double-stained cells appeared as brown nuclei with red dots (Fig. 3a inset). Furthermore, the known interaction of Sox2 with the cell cycle regulator cyclin D (Han et al. 2014; Swistowska et al. 2019) seem to indicate a direct connection between the yielded Sox2 signals and cell proliferation.

### Cytoplasmic immunosignals

In addition to the specific nuclear immunosignals described in the manufacturer’s information, some neurons revealed Sox2 (Fig. 3a) and pRb immunostaining also in the cytoplasm, but according to the isotype-control results, only the cytoplasmic signals of Sox2 proved to be specific.

Sox2 and other Sox proteins are known to control their activity through nucleocytoplasmic shuttling in development (Smith and Koopman 2004), where Sox2 moves from nucleus to cytoplasm most likely in response to differentiation signals (Baltus et al. 2009). Thus, the cytoplasmic signals within the mature neurons of our cats may support our result-based presumption of feline reactivated terminally differentiated neurons.

To emphasize, irrespective of age, sex, and illness, and regardless of their parvovirus-antigen status, there were no differences concerning the expression patterns of pRb and Sox2 as we yielded specific immunosignals for both antibodies in all cats.

### Cell cycle reactivation due to general diseases?

Our study highlights that parvovirus seems not to be the sole driving element, why post-mitotic feline neurons leave G0. Comparing the parvovirus-antigen negative brains to the brains proven positive, we could not find any differences in the expression pattern; even FIP and FeLV infections in some of the cats investigated had no influence on the expression levels of Sox2 and pRb. Our data therefore agree with our previously demonstrated CnD1, PCNA, and Ki67 expression in mature neurons of parvovirus-antigen negative feline brains (Gruber et al. 2004) and confirm the thesis of a parvovirus-independent cell cycle re-entry.

In almost all cats of group B there was acute panleukopenia, so detection of residues of former parvovirus infections within the neurons seems highly unlikely, the more, as there was no evidence of peri/prenatal parvovirus infection due to an unaltered cerebellum in all cats of group B. In this regard, parvovirus can be excluded as the causative element for neuronal cell cycle re-entry in cats, and most likely only “uses” the activated mature neurons to replicate within those post-mitotic fixed and terminally differentiated neurons.

Severe diseases are known to account for systemic impacts, and neurons are known to be exceptionally susceptible to DNA damage due to oxidative stress. As previously described, DNA damage response of post-mitotic neurons seems to lead either to cell cycle-related DNA repair or to apoptosis-induced neuronal death (Andrusiak et al. 2012; Kruman 2004; Schwartz et al. 2007; Zhang et al. 2020).

Hence, although the parvovirus-antigen negative brains revealed no morphological alterations or diseases (besides two cats with cerebral FIP), the bodies of those cats did—as did the bodies of the cats with parvovirus-antigen positive neurons. So probably, instead of neuronal parvovirus infection, the serious illnesses account for the activation of the neuronal cell cycle machinery due to oxidative stress-related neuronal DNA damage in those cats.

## Conclusions

To conclude, this study strengthens the hypotheses of cell cycle reactivation in mature feline neurons, however, not solely driven by parvovirus. It remains to be clarified, whether the cell cycle re-entry of those neurons is activated due to DNA damage-initiated apoptosis or is initiated to contribute to DNA repair and neuronal survival.

## Data Availability

The datasets generated during the current study are available from the corresponding author on reasonable request.
